# Texture analysis using Horadam polynomial coefficient estimate for the class of Sakaguchi kind function

**DOI:** 10.1038/s41598-023-41734-w

**Published:** 2023-09-02

**Authors:** H. Priya, B. Sruthakeerthi

**Affiliations:** Department of Mathematics, School of Advanced Sciences, Vellore Institute of Technology, Chennai Campus, Chennai, India

**Keywords:** Engineering, Mathematics and computing

## Abstract

This present work provides the initial co-efficient bounds of the function *f*(*z*) which is defined in open unit disk $\mathbb {D}$. We introduced bi-univalent class $\mathcal {C}_{\Sigma }(\lambda , t,\nu )$. Making use of Horadam polynomials $h_{n}(\nu )$ and the generating function $\Pi (\nu ,z)$, we estimated the bounds of $a_{2}$ and $a_{3}$ for the given function to be in the defined class. Moreover Fekete-Szego inequalities are calculated. In addition to all the results obtained mathematically, we provide application for Horadam polynomial in computer vision.

## Introduction

A function *f* belonging to the class $\mathcal {A}$ is analytic in open unit disk $\mathbb {D}=\{z \in \mathbb {C}: |z|<1 \}$. Where the class $\mathcal {A}$ contains all analytic functions of the form

1 $$\begin{aligned} f(z)=z+\sum _{n=2}^{\infty } a_{n}z^{n}. \end{aligned}$$

While a subclass $\mathcal {S}$ contains all univalent functions in open unit disk $\mathbb {D}$. Every function $f\in \mathcal {S}$ has an inverse which is defined as $f^{-1}((z))= z,~(z\in \mathbb {D})$ and $f(f^{-1}(w))=w$, $(|w| <r_{0}(f); r_{0} \ge \frac{1}{4})$,

where

2 $$\begin{aligned} f^{-1}(w)=w-a_{2}w^{2}+(2a_{2}^{2}-a_{3})w^{3}-(5a^{3}_{2}-5a_{2}a_{3}+a_{4})w^{4}+. . . . \end{aligned}$$

A bi-univalent function is a complex-valued function that is both bijective and univalent in the open unit disk of the complex plane. In other words, a bi-univalent function is a function that maps the open unit disk onto a bijective image in the complex plane, such that the function is one-to-one and has a single-valued inverse function in the open unit disk.

More formally, a function f(z) is bi-univalent in the open unit disk if and only if:

- *f*(*z*) is analytic and injective in the open unit disk, i.e., for any two distinct points $z_{1}$ and $z_{2}$ in the open unit disk, $f(z_{1})$ does not equal $f(z_{2})$.
- *f*(*z*) maps the open unit disk onto a bijective image, i.e., for every point *w* in the image, there exists a unique point *z* in the open unit disk such that $f(z) = w$.
- The inverse function $f^{-1}(z)$ is also analytic and injective in the image of *f*(*z*).

Bi-univalent functions have been studied extensively in complex analysis and geometric function theory due to their properties and applications in various fields such as mathematical physics, engineering, and image processing. One of the fundamental results related to bi-univalent functions is the Bieberbach conjecture, which states that the Taylor coefficients of a bi-univalent function in the open unit disk satisfy certain inequalities. This conjecture was proved by Louis de Branges in 1985, and it has many important consequences in complex analysis. Examples of bi-univalent functions include the Koebe function, the Bessel function, and various subclasses of bi-univalent functions such as starlike and convex functions.

Precisely if a function $f\in \mathcal {A}$ and its inverse $f^{-1}$ are univalent in $\mathbb {D}$ then the function *f* is said to be bi-univalent in $\mathbb {D}$. Let $\Sigma$ is the symbol of the class of bi-univalent functions in $\mathbb {D}$ of the form (1)

### Definition 1

Starlike functions^1^ A simple connected domain $\mathbb {D}\subset \mathbb {C}$ is supposed to be starlike with regard to $w_0 \in \mathbb {D}$, if there exists a line segment which joins $w_0$ to any other point of $\mathbb {D}$ and it completly lies in $\mathbb {D}$.

Suppose $u \in \mathcal {S}$ is claimed to be star-like function relevant to the origin, if *u* maps the unit disk $\mathbb {U}$ onto a star-like domain in view of the origin. The family $S^{*}$ denotes the collection of all star-like functions in view of origin.

$$\begin{aligned} \mathcal {S}^*=\left\{ f \in \mathcal {S}\ :\ \frac{z f^\prime (z)}{f(z)} \in \mathcal {P}\right\} . \end{aligned}$$

Equivalently,

$$\begin{aligned} f \in \mathcal {S}^* \ \ \ \text {iff} \ \ \ \Re \left\{ \frac{z f^\prime (z)}{f(z)}\right\} >0. \end{aligned}$$

### Definition 2

Convex functions (Goodman 1983) In a complex plane $\mathbb {C}$, a domain $\mathbb {D}$ is supposed to be convex if it is star-like concerning each of its points, (i.e) if every pair of points of $\mathbb {D}$ can be joined by a line segment, which completly lies in $\mathbb {D}$.

A function $u \in \mathcal {S}$ is claimed to be a convex function if *u* maps the unit disk $\mathbb {U}$ onto a convex domain. The family $\mathcal {C}$ indicates the collection of all convex functions.

$$\begin{aligned} \mathcal {C}=\left\{ f \in \mathcal {S}\ \ : \ \ 1+\frac{z f^{\prime \prime }(z)}{f^\prime (z)} \in \mathcal {P}\right\} . \end{aligned}$$

Equivalently,

$$\begin{aligned} f \in \mathcal {C}\ \ \ \text {if and only if} \ \ \ \Re \left\{ 1+\frac{z f^{\prime \prime }(z)}{f^\prime (z)}\right\} >0. \end{aligned}$$

Recently Srivastava et al.^2^ have studied analytic and bi-univalent functions. Many authors have studied and provided various subclasses of bi-univalent functions and fixed the initial co-efficients $|a_{2}|$ and $|a_{3}|$ [see^3–8^ and^9^].

For two analytic functions *f*(*z*) and *g*(*z*) , if *f*(*z*) subordinate to *g*(*z*) and it can be written as $f\prec g$ if there exists a Schwartz function *w* with $|w(z)|<1$ and $w(0)=0$ such that $f(z)=g(w(z))$. It is well known that the function *g* is univalent in $\mathbb {D}$ then $f\prec g$ $\Leftrightarrow$ $f(0)=g(0)$ and $f(\mathbb {D})\subset g(\mathbb {D}).$

The Horadam polynomials $h_{n}(\nu )$ are defined by the following relation (see^10^),

$$\begin{aligned} h_{n}(\nu )=e_{1}\nu h_{n-1}(\nu )+e_{2}h_{n-2}(\nu ) \quad (\nu \in \mathbb {R}, n\in \mathbb {N}\ge 2) \end{aligned}$$

with

3 $$\begin{aligned} h_{1}(\nu )=a ~~ \text{ and } ~~ h_{2}(\nu )=b\nu \end{aligned}$$

where $a,b,e_{1},e_{2}$ are real constants.

The generating function of the Horadam polynomials $h_{n}(\nu )$ (see^11^) is given by

4 $$\begin{aligned} \Pi (\nu , z)=\sum _{n=1}^{\infty }h_{n}(\nu )z^{n-1} =\frac{a+(b-ae_{1})\nu z}{1-e_{1}\nu z-e_{2} z^{2}}. \end{aligned}$$

Frasin^12^ investigated the inequalities of co-efficient for certain classes of generalized Sakaguchi type function *f* satisfying the following geometrical property

5 $$\begin{aligned} \Re \left\{ \frac{(s-t)z(f^{'}(z))}{f(sz)-f(tz)} \right\} >\alpha , \end{aligned}$$

*s*, *t* are complex numbers with $s\ne t$ and $0 \le \alpha <1$.

For the fixed values of $a,b,e_{1}$ and $e_{2}$ the Horadam polynomial $h_{n}(\nu )$ provides various types of polynomials, from these, we have listed a few here (see^13– 21^):

1. When $a=2 ~~ \& ~~ b=e_{1}=e_{2}=1$, we have the Lucas polynomial $L_{n}(\nu )$
2. When $a=b=e_{1}=e_{2}=1$, we have the Fibonacci polynomial $F_{n}(\nu )$
3. When $a=b=e_{1}=2~~ \& ~~ e_{2}=1$, we arrive the pell-Lucas polynomials $Q_{n}(\nu )$
4. When $a=e_{2}=1 ~~ \& ~~ b=e_{2}=2$, we reaches the pell polynomial $P_{n}(\nu )$
5. When $a=2, b=e_{1}=2$ and $e_2=-1$, we have the chebyshev polynomials $U_{n}(\nu )$ of the second kind.
6. When $a=b=1$ and $e_{1}=2, e_{2}=-1$, we get the chebyshev polynomials $T_{n}(\nu )$ of the first kind.

### Definition 3

A function $f(z)=z+\sum _{n=2}^{\infty } a_{n}z^{n}$ $\in$ $\Sigma$ is said to be in the class $\mathcal {C}_{\Sigma }(\lambda , t,\nu )$ if it satisfies the following subordination conditions which are follows,

$$\begin{aligned} \frac{(1-t)\left[ \lambda z^{3}f^{\prime \prime \prime }(z)+(1+2\lambda )z^{2}f^{\prime \prime }(z)+zf^{\prime }(z)\right] }{\lambda z^{2}\left[ f^{\prime \prime }(z)-t^{2}f^{\prime \prime }(tz)\right] +z\left[ f^{\prime } (z)-tf^{\prime }(tz)\right] } \prec \Pi (\nu ,z)+1-a \end{aligned}$$

and

$$\begin{aligned} \frac{(1-t)\left[ \lambda \omega ^{3}r^{\prime \prime \prime }(\omega )+(1+2\lambda )\omega ^{2}r^{\prime \prime }(\omega )+\omega r^{\prime }(\omega )\right] }{\lambda \omega ^{2}\left[ r^{\prime \prime }(\omega )-t^{2}r^{\prime \prime }(t\omega )\right] +\omega \left[ r^{\prime } (\omega )-tr^{\prime }(t\omega )\right] } \prec \Pi (\nu ,\omega )+1-a \end{aligned}$$

where $0 \le \lambda \le 1$, $|t| \le 1$ with $t\ne 1$, $\nu \in \mathbb {R}$ and *a* is a real constant, the function $r=f^{-1}$ is given by (2).

**Special Case:1** For $t=0$ the class $\mathcal {C}_{\Sigma }(\lambda ,t,\nu )$ is reduced to $\mathcal {C}_{\Sigma }(\lambda ,\nu )$ satisfying the following conditions

$$\begin{aligned} \frac{\lambda z^{3}f^{\prime \prime \prime }(z)+(1+2\lambda )z^{2}f^{\prime \prime }(z)+zf^{\prime }(z)}{\lambda z^{2}f^{\prime \prime }(z)+zf^{\prime } (z)} \prec \Pi (\nu ,z)+1-a \end{aligned}$$

and

$$\begin{aligned} \frac{\lambda \omega ^{3}r^{\prime \prime \prime }(\omega )+(1+2\lambda )\omega ^{2}r^{\prime \prime }(\omega )+\omega r^{\prime }(\omega )}{\lambda \omega ^{2}r^{\prime \prime }(\omega )+\omega r^{\prime } (\omega )} \prec \Pi (\nu ,\omega )+1-a. \end{aligned}$$

**Special Case:2** For the case $\lambda =0$ in the class $\mathcal {C}_{\Sigma }(\lambda ,t,\nu )$ reduces to the class $\mathcal {C}_{\Sigma }(t,\nu )$ satisfying the following conditions,

$$\begin{aligned} \frac{(1-t)\left[ z^{2}f^{\prime \prime }(z)+z f^{\prime }(z)\right] }{z\left[ f^{\prime }(z)-tf^{\prime }(tz)\right] } \prec \Pi (\nu ,z)+1-a \end{aligned}$$

and

$$\begin{aligned} \frac{(1-t)\left[ \omega ^{2}r^{\prime \prime }(\omega )+\omega r^{\prime }(\omega )\right] }{\omega \left[ r^{\prime }(\omega )-tr^{\prime }(t\omega )\right] } \prec \Pi (\nu ,\omega )+1-a. \end{aligned}$$

## Main results

### Theorem 4

A function $f(z)=z+\sum _{n=2}^{\infty } a_{n}z^{n}$ $\in$ $\mathcal {A}$ be in the class $\mathcal {C}_{\Sigma }(\lambda , t, \nu )$, then

$$\begin{aligned}&|a_{2}|\le \frac{|b \nu |\sqrt{|b \nu |}}{\sqrt{\left| 2\left\{ 4(u_{2}-2)\{u_{2}b^{2}\nu ^{2}-(pb\nu ^{2}+qa)(u_{2}-2)(1+\lambda )\} -3(u_{3}-3)b^{2}\nu ^{2}\right\} \right| }} \nonumber \\&|a_{3}|\le \frac{|b\nu |}{3(1+2\lambda )(3-u_{3})} +\frac{|b^{2}v^{2}|}{ 4(1+\lambda )^{2}(2-u_{2})^{2}} \end{aligned}$$

where

$$\begin{aligned} u_{n}=\frac{1-t^{n}}{1-t}, ~~ n\in \mathbb {N}\end{aligned}$$

### Proof

Let $$f \in \mathcal {C}_{\Sigma }(\lambda , t,\nu )$$.

Let the function $\Omega (z)$ and $\Psi (w)$ are analytic in $\mathbb {D}$. $\Omega , \Psi :\mathbb {D}\rightarrow \mathbb {D}$ given by

6 $$\begin{aligned} \Omega (z)=\Omega _{1}z+\Omega _{2}z^{2}+\Omega _{3}z^{3}+. . . . (z \in \mathbb {D}) \end{aligned}$$

7 $$\begin{aligned} \Psi (w)=\Psi _{1}w+\Psi _{2}w^{2}+\Psi _{3}w^{3}+. . . . (w \in \mathbb {D}) \end{aligned}$$

with

$$\begin{aligned} \Omega (0)=\Psi (0)=0, |\Omega (z)|<1, |\Psi (z)|<1,~~ z,w \in \mathbb {D}\end{aligned}$$

such that

$$\begin{aligned} \frac{(1-t)\left[ \lambda z^{3}f^{\prime \prime \prime }(z)+(1+2\lambda )z^{2}f^{\prime \prime }(z)+zf^{\prime }(z)\right] }{\lambda z^{2}\left[ f^{\prime \prime }(z)-t^{2}f^{\prime \prime }(tz)\right] +z\left[ f^{\prime } (z)-tf^{\prime }(tz)\right] } = \Pi (\nu ,\Omega (z))+1-a \end{aligned}$$

and

$$\begin{aligned} \frac{(1-t)\left[ \lambda \omega ^{3}r^{\prime \prime \prime }(\omega )+(1+2\lambda )\omega ^{2}r^{\prime \prime }(\omega )+\omega r^{\prime }(\omega )\right] }{\lambda \omega ^{2}\left[ r^{\prime \prime }(\omega )-t^{2}r^{\prime \prime }(t\omega )\right] +\omega \left[ r^{\prime } (\omega )-tr^{\prime }(t\omega )\right] } = \Pi (\nu ,\Psi (\omega ))+1-a \end{aligned}$$

which is equivalently,

8 $$\begin{aligned}&\frac{(1-t)\left[ \lambda z^{3}f^{\prime \prime \prime }(z)+(1+2\lambda )z^{2}f^{\prime \prime }(z)+zf^{\prime }(z)\right] }{\lambda z^{2}\left[ f^{\prime \prime }(z)-t^{2}f^{\prime \prime }(tz)\right] +z\left[ f^{\prime } (z)-tf^{\prime }(tz)\right] } \nonumber \\&~~~~~~~~~~~~~~~~~~~~~~~~~~~~~~~~~= 1+h_{1}(\nu )-a+h_{2}(\nu ) \Omega (z)+h_{3} \Omega ^{2}(z)+. . . . \end{aligned}$$

and

9 $$\begin{aligned}&\frac{(1-t)\left[ \lambda \omega ^{3}r^{\prime \prime \prime }(\omega )+(1+2\lambda )\omega ^{2}r^{\prime \prime }(\omega )+\omega r^{\prime }(\omega )\right] }{\lambda \omega ^{2}\left[ r^{\prime \prime }(\omega )-t^{2}r^{\prime \prime }(t\omega )\right] +\omega \left[ r^{\prime } (\omega )-tr^{\prime }(t\omega )\right] } \nonumber \\&~~~~~~~~~~~~~~~~~~~~~~= 1+h_{1}(\nu )-a+h_{2}(\nu ) \Psi (w)+h_{3} \Psi ^{2}(w)+. . . . \end{aligned}$$

Joining (6), (7) ,(8) and (9), gives

10 $$\begin{aligned}&\frac{(1-t)\left[ \lambda z^{3}f^{\prime \prime \prime }(z)+(1+2\lambda )z^{2}f^{\prime \prime }(z)+zf^{\prime }(z)\right] }{\lambda z^{2}\left[ f^{\prime \prime }(z)-t^{2}f^{\prime \prime }(tz)\right] +z\left[ f^{\prime } (z)-tf^{\prime }(tz)\right] } \nonumber \\&~~~~~~~~~~~~~~~~~~~~~~~~~~~~~~~~~=1+h_{2}(\nu )\Omega _{1}z+\left[ h_{2}(\nu )\Omega _{2}+h_{3}(\nu )\Omega _{1}^{2}\right] z^{2}+.... \end{aligned}$$

and

11 $$\begin{aligned}&\frac{(1-t)\left[ \lambda \omega ^{3}r^{\prime \prime \prime }(\omega )+(1+2\lambda )\omega ^{2}r^{\prime \prime }(\omega )+\omega r^{\prime }(\omega )\right] }{\lambda \omega ^{2}\left[ r^{\prime \prime }(\omega )-t^{2}r^{\prime \prime }(t\omega )\right] +\omega \left[ r^{\prime } (\omega )-tr^{\prime }(t\omega )\right] } \nonumber \\&~~~~~~~~~~~~~~~~~~~~~~~~~~~~~~~=1+h_{2}(\nu )\Psi _{1}w+\left[ h_{2}(\nu )\Psi _{2}+h_{3}(\nu )\Psi _{1}^{2}\right] w^{2}+.... \end{aligned}$$

we know that if $|\Omega (z)|<1$ and $|\Psi (w)|<1$, $~~ z, w \in \mathbb {D}$

12 $$\begin{aligned} \text {then} ~~~|\Omega _{i}|\le 1~~~ \text {and}~~~ |\Psi _{i}|\le 1 ~~~ \forall i\in \mathbb {N}. \end{aligned}$$

Equate like co-efficients in (10) and (11), and simplifying,

we get

13 $$\begin{aligned}&2a_{2}(1+\lambda )(2-u_{2})=h_{2}(\nu )\Omega _{1} \end{aligned}$$

14 $$\begin{aligned}&3a_{3}(1+2\lambda )(3-u_{3})+4a^{2}_{2}u_{2}(1+\lambda )^{2}(u_{2}-2)=h_{2}(\nu )\Omega _{2}+h_{3}(\nu )\Omega _{1}^{2} \end{aligned}$$

15 $$\begin{aligned}&-2a_{2}(1+\lambda )(2-u_{2})=h_{2}(\nu )\Psi _{1} \end{aligned}$$

16 $$\begin{aligned}&3(1+2\lambda )(2a_{2}^{2}-a_{3})(3-u_{3})+4a^{2}_{2}u_{2}(1+\lambda )(u_{2}-2)=h_{2}(\nu )\Psi _{2}+h_{3}(\nu )\Psi _{1}^{2} \end{aligned}$$

By (13) and (15) we see that

17 $$\begin{aligned} \Omega _{1}=-\Psi _{1} \end{aligned}$$

Squaring and adding (13) and (15), we have

18 $$\begin{aligned} 2\left[ 4(1+\lambda )^{2}(2-u_{2})^{2}\right] a_{2}^{2}=h_{2}^{2}(\nu )(\Omega _{1}^{2}+\Psi _{1}^{2}) \end{aligned}$$

By adding (14) and (16) , we have

19 $$\begin{aligned} 2\left[ 3(3-u_{3})+4u_{2}(u_{2}-2)\right] a_{2}^{2}=h_{2}(\nu )(\Omega _{2}+\Psi _{2})+h_{3}(\nu )(\Omega _{1}^{2}+\Psi _{1}^{2}) \end{aligned}$$

By Making use of (15) it is reduced that

20 $$\begin{aligned} a_{2}^{2}=\frac{h_{2}^{3}(\nu )(\Omega _{2}+\Psi _{2})}{2\left\{ 4(u_{2}-2)\{u_{2} h_{2}^{2}(\nu )-h_{3}(\nu )(u_{2}-2)(1+\lambda )\}-3(u_{3}-3)h_{2}^{2}(\nu )\right\} } \end{aligned}$$

using equations (3) and (12) in (20), we obtain

$$\begin{aligned}&|a_{2}|\le \frac{|b \nu |\sqrt{|b \nu |}}{\sqrt{\left| 2\left\{ 4(u_{2}-2)\{u_{2}b^{2}\nu ^{2} -(pb\nu ^{2}+qa)(u_{2}-2)(1+\lambda )\}-3(u_{3}-3)b^{2}\nu ^{2}\right\} \right| }} \end{aligned}$$

Difference between (14) and (16), follows that

21 $$\begin{aligned} 6(1+2\lambda )(3-u_{3})(a_{3}-a_{2}^{2}) =h_{2}(\nu )(\Omega _{2}-\Psi _{2}) \end{aligned}$$

By (17) and (18) we have from (21)

$$\begin{aligned} a_{3}=\frac{h_{2}(\nu )(\Omega _{2}-\Psi _{2})}{6(1+2\lambda )(3-u_{3})}+ \frac{h_{2}^{2}(\nu )(\Omega _{1}^{2}+\Psi _{1}^{2})}{8(1+\lambda )^{2}(2-u_{2})^{2}} \end{aligned}$$

Using (3), we get

$$\begin{aligned} |a_{3}|\le \frac{|b\nu |}{3(1+2\lambda )(3-u_{3})}+\frac{|b^{2}v^{2}|}{4(1+\lambda )^{2}(2-u_{2})^{2}} \end{aligned}$$

This completes the proof of the theorem (4). $\square$

If we take $\lambda =0$ in Theorem 4, we obtain the following corollary,

### Corollary 5

A function $f \in \mathcal {A}$ and of the form (1) is in the class $\mathcal {C}_{\Sigma }(t,\nu )$, then

$$\begin{aligned}&|a_{2}|\le \frac{|b \nu |\sqrt{|b \nu |}}{\sqrt{\left| 2\left\{ 4(u_{2}-2)\{u_{2}b^{2}\nu ^{2} -(pb\nu ^{2}+qa)(u_{2}-2)\}-3(u_{3}-3)b^{2}\nu ^{2}\right\} \right| }} \nonumber \\&|a_{3}|\le \frac{|b\nu |}{3(3-u_{3})}+\frac{|b^{2}v^{2}|}{4(2-u_{2})^{2}} \end{aligned}$$

### Remark 6

When $t=0$,The class $\mathcal {C}_{\Sigma }(t,\nu )$ is reduced to the class $\mathcal {C}_{\Sigma }(\nu )$.

### Theorem 7

For any complex number $\tau$, Let $f(z)=z+\sum _{n=2}^{\infty } a_{n}z^{n}$ $\in$ $\mathcal {A}$ be in the class $\mathcal {C}_{\Sigma }(\lambda , t, \nu )$ then

$$\begin{aligned} |a_{3}-\tau a_{2}^{2}| \le {\left\{ \begin{array}{ll} \frac{ |h_{2}(\nu )|}{ 3(1+2\lambda )(3-u_{3})} , &{} |\tau -1|\le \left| \frac{\begin{array}{r}\left\{ \left[ 3(1+2\lambda )(3-u_{3})-2u_{2}(1+\lambda )^{2}(2-u_{2})\right] b^{2}\nu ^{2} \right. \\ \left. -4(pb\nu ^{2}+qa)(1+\lambda )^{2}(2-u_{2})^{2}\right\} \end{array}}{\begin{array}{r} 3b^{2}\nu ^{2}(1+2\lambda )(3-u_{3})\end{array}}\right| \\ 2|h_{2}(\nu )|\phi (\nu ,\tau )|, &{} |\tau -1|\ge \left| \frac{\begin{array}{r}\left\{ \left[ 3(1+2\lambda )(3-u_{3})-2u_{2}(1+\lambda )^{2}(2-u_{2})\right] b^{2}\nu ^{2} \right. \\ \left. -4(pb\nu ^{2}+qa)(1+\lambda )^{2}(2-u_{2})^{2}\right\} \end{array}}{\begin{array}{r}3b^{2}\nu ^{2} (1+2\lambda )(3-u_{3})\end{array}}\right| \end{array}\right. } \end{aligned}$$

where

$$\begin{aligned} \phi (\tau ,\nu )=\frac{(1-\tau )h_{2}^{2}(\nu )}{2\left\{ \left[ 3(1+2\lambda )(3-u_{3}) -2u_{2}(1+\lambda )^{2}(2-u_{2})\right] h_{2}^{2}(\nu )-4h_{3}(\nu )(1+\lambda )^{2}(2-u_{2})^{2}\right\} } \end{aligned}$$

and

$$\begin{aligned} u_{n}=\frac{1-t^{n}}{1-t}, ~~ n\in \mathbb {N}\end{aligned}$$

### Proof

From (20) and (21)

$$\begin{aligned} a_{3}- a_{2}^2&=\frac{h_{2}(\nu )(\Omega _{2}-\Psi _{2})}{6(1+2\lambda )(3-u_{3}) } \nonumber \\ a_{3}- \tau a_{2}^2&=\frac{h_{2}(\nu )(\Omega _{2}-\Psi _{2})}{6(1+2\lambda )(3-u_{3})}+a_{2}^{2}- \tau a_{2}^{2} \nonumber \\&=\frac{h_{2}(\nu )(\Omega _{2}-\Psi _{2})}{6(1+2\lambda )(3-u_{3})}+(1-\tau )a_{2}^{2} \nonumber \\ a_{3}- \tau a_{2}^2&=\frac{h_{2}(\nu )(\Omega _{2}-\Psi _{2})}{6(1+2\lambda )(3-u_{3})}+ \frac{(1-\tau )h_{2}^{3}(\nu )(\Omega _{2}+\Psi _{2})}{\begin{array}{r} 2\left\{ \left[ 3(1+2\lambda )(3-u_{3})-2u_{2}(1+\lambda )^{2}(2-u_{2})\right] h_{2}^{2}(\nu ) \right. \\ \left. -4h_{3}(\nu )(1+\lambda )^{2}(2-u_{2})^{2}\right\} \end{array}} \nonumber \\&=h_2(\nu )\bigg [\frac{(\Omega _{2}-\Psi _{2})}{6(1+2\lambda )(3-u_{3})} +\frac{(1-\tau )h_{2}^{2}(\nu )(\Omega _{2}+\Psi _{2})}{\begin{array}{r} 2\left\{ \left[ 3(1+2\lambda )(3-u_{3})-2u_{2}(1+\lambda )^{2}(2-u_{2})\right] h_{2}^{2}(\nu ) \right. \\ \left. -4h_{3}(\nu )(1+\lambda )^{2}(2-u_{2})^{2}\right\} \end{array}}\bigg ] \nonumber \\&=h_{2}(\nu )\bigg [\frac{(\Omega _{2}-\Psi _{2})}{6(1+2\lambda )(3-u_{3})}+\phi (\tau ,\nu )(\Omega _{2}+\Psi _{2})\bigg ] \end{aligned}$$

where

$$\begin{aligned} \phi (\tau ,\nu )&=\frac{(1-\tau )h_{2}^{2}(\nu )}{2\left\{ \left[ 3(1+2\lambda )(3-u_{3}) -2u_{2}(1+\lambda )^{2}(2-u_{2})\right] h_{2}^{2}(\nu )-4h_{3}(\nu )(1+\lambda )^{2}(2-u_{2})^{2}\right\} }\\&=h_{2}(\nu )\bigg [\frac{\Omega _{2}}{6(1+2\lambda )(3-u_{3})}-\frac{\Psi _{2}}{6(1+2\lambda )(3-u_{3})} +\phi (\tau ,\nu )\Omega _{2}+\phi (\tau ,\nu )\Psi _{2})\bigg ]\\&=h_{2}(\nu )\left\{ \left( \phi (\tau ,\nu )+\frac{1}{6(1+2\lambda )(3-u_{3})}\right) \Omega _{2} +\left( \phi (\tau ,\nu )-\frac{1}{6(1+2\lambda )(3-u_{3})}\right) \Psi _{2}\right\} \end{aligned}$$

Hence, we have

$$\begin{aligned} |a_{3}-\tau a_{2}^{2}| \le {\left\{ \begin{array}{ll} \frac{ |h_{2}(\nu )|}{ 3(1+2\lambda )(3-u_{3})} , &{} 0 \le |\phi (\tau ,\nu )|\le \frac{1}{6(1+2\lambda )(3-u_{3})} \\ 2|h_{2}(\nu )|\phi (\nu ,\tau )|, &{} |\phi (\tau ,\nu )|\ge \frac{1}{6(1+2\lambda )(3-u_{3})} \end{array}\right. } \end{aligned}$$

$$\square$$

For $\lambda =0$ and $t=0$ in Theorem (7), the following co-efficient estimates is obtained.

### Corollary 8

A function $f \in \mathcal {A}$ and of the form (1) is in the class $\mathcal {C}_{\Sigma }(\nu )$, then

$$\begin{aligned} |a_{2}|&\le \frac{|b\nu |\sqrt{|b\nu |}}{\sqrt{|2b^{2}\nu ^{2}-4(pb\nu ^{2}+qa)|}}\nonumber \\ |a_{3}|&\le \frac{|b\nu |}{6}+\frac{b^{2}v^{2}}{4} \nonumber \\ |a_{3}-\tau a_{2}^{2}|&\le {\left\{ \begin{array}{ll} \frac{ \left| b\nu \right| }{6} , &{} |\tau -1|\le \frac{\left| 2b^{2}\nu ^{2}-4(pb\nu ^{2}+qa) \right| }{6|b^{2}\nu ^{2}|}\\ \frac{|b ^{3} \nu ^{3}| |\tau -1|}{|2b^{2}\nu ^{2}-4(pb\nu ^{2}+qa)|}, &{} |\tau -1|\ge \frac{\left| 2b^{2}\nu ^{2}-4(pb\nu ^{2}+qa) \right| }{6|b^{2}\nu ^{2}|} \end{array}\right. } \end{aligned}$$

## Application

The Horadam polynomial is an illustration of a mathematical sequence used in texture analysis and image processing. The Horadam polynomial, a specific kind of polynomial sequence, can be used to perform procedures like filtering and resampling. In image processing and computer vision, scale-space representations of images can be created and modified using the Horadam polynomial. This polynomial can be used for edge identification, texture analysis, and multi-scale image analysis. About texture analysis, the Horadam polynomial has been used for feature extraction, segmentation, and picture denoising. This technique can be used to analyze the statistical properties of textures, including the distribution of gray levels and the spatial organization of textures.

This article focuses on texture analysis utilizing canny images by convoluting the calculated co-efficient values to the image pixels using masking at eight different angles and displaying the best result.

**Figure 1:**
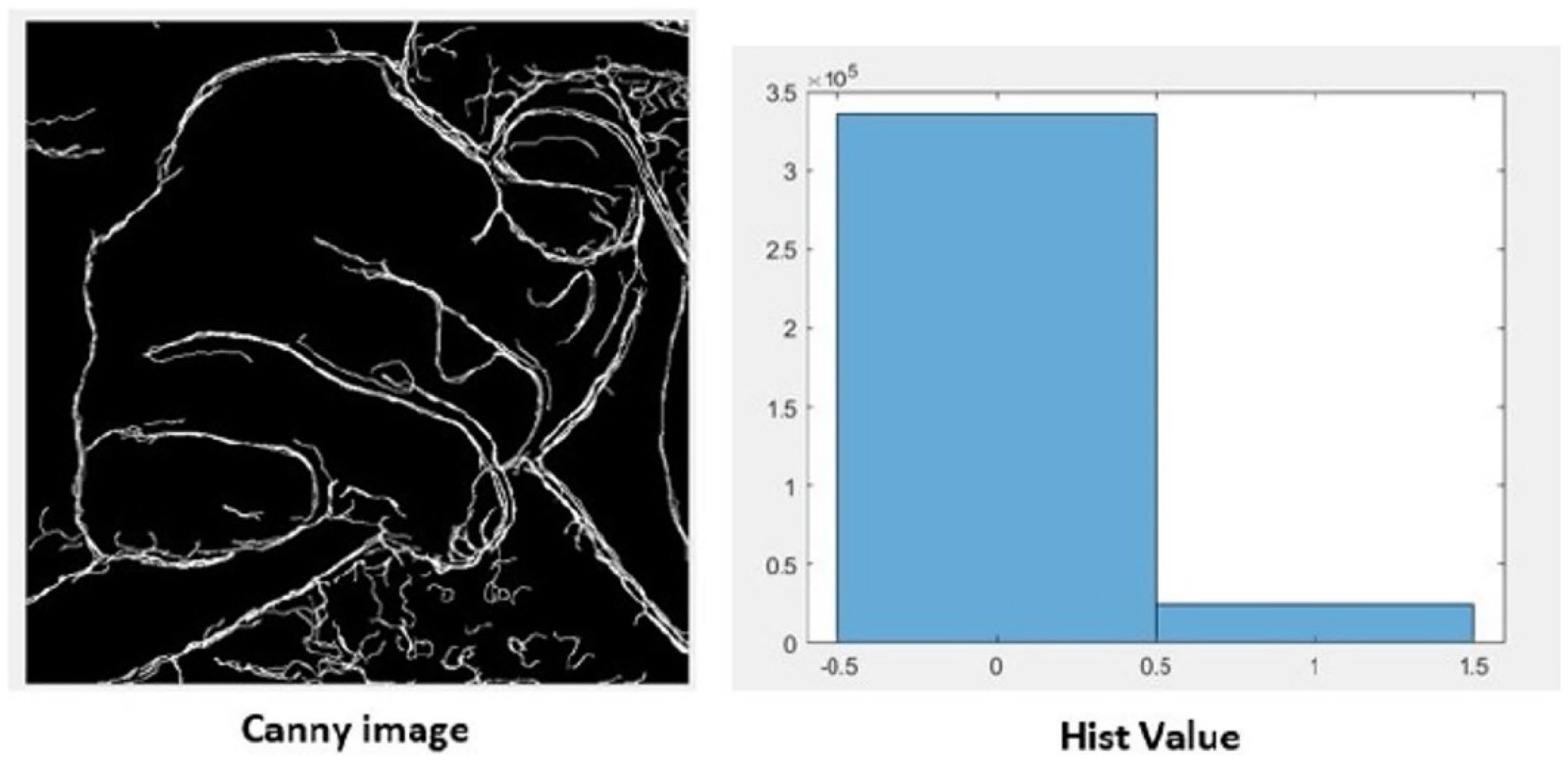
Canny Image of the closed palm using Edge detection algorithm.

The image used to comprehend is a closed palm image of resolution 600$\times$600. The original image and its corresponding Hist Value are shown in the Fig. 1.

**Figure 2:**
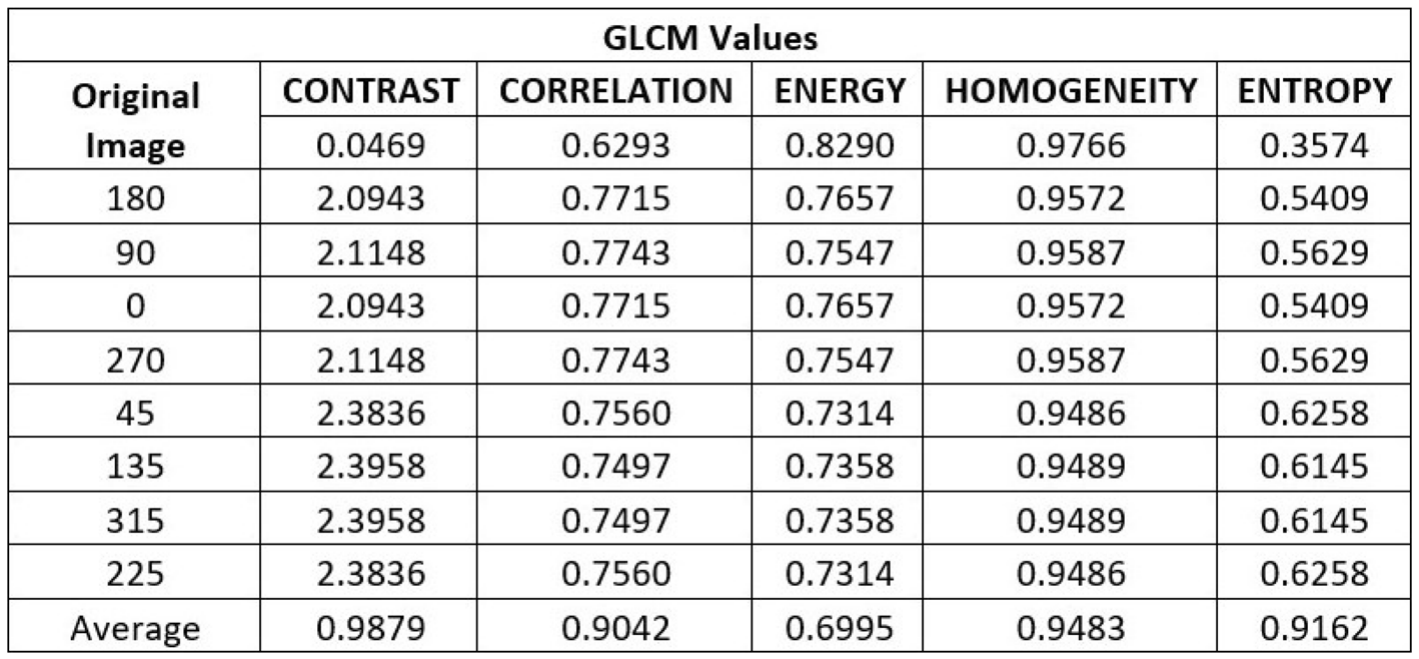
Representation of Gray-Level Co-Occurrence Matrix (GLCM) values obtained by MATLAB.

## Elucidation

**Figure 3:**
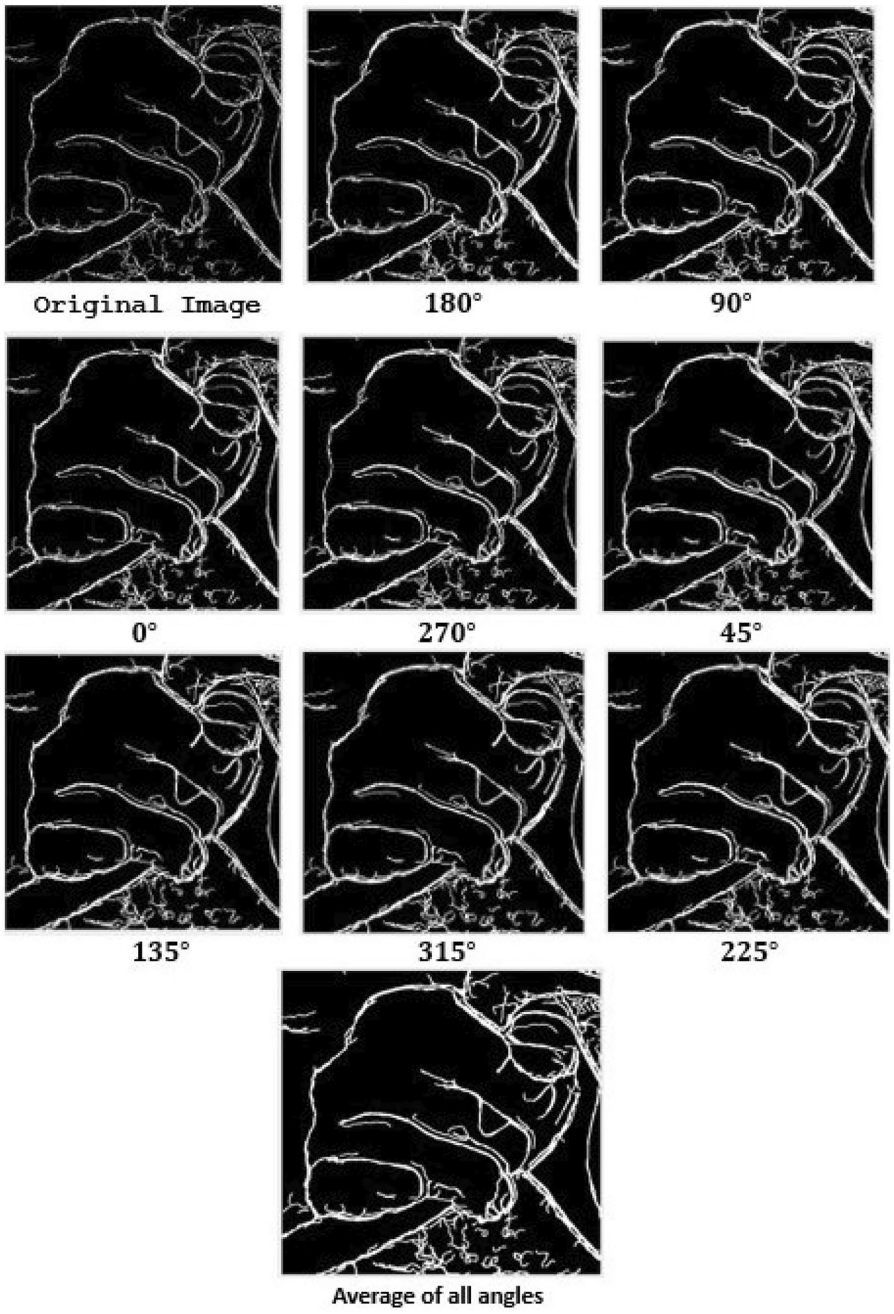
The original Image is the canny image of a closed palm, using the quality metrics, contrast, correlation, entropy, energy, and homogeneity. Above images show the canny image of the same at angles of $180^{\circ }, 90^{\circ }, 0^{\circ }, 270^{\circ }, 45^{\circ }, 135^{\circ }, 315^{\circ }, \text {and} 255^{\circ }$, respectively, with increased contrast, correlation, and entropy and decreased energy and homogeneity. The final figure shows a canny representation of the average of the aforementioned angles along with the relevant metrics.

Figure 2 displays the GLCM values for the canny image at various angles. The tabulation also shows that the Horadam polynomial examines the texture of the supplied image and that the values are also quite good for the contrast, correlation, energy, homogeneity, and entropy requirements. The suggested Texture method thus yields superior outcomes. The results of the image taken at various angles are shown in Fig. 3.

## Conclusion

The goal of the manuscript was to link geometric function theory and image processing, specifically texture analysis. The image selected is solely a canny image, and it is treated using the outcomes of the work’s mathematical analysis, the study is justified by the obtained values of the metrics utilised, such as contrast, correlation, entropy, energy, and homogeneity. The work also sheds lime light to work with coloured images and investigate various image-processing techniques like enhancement, sharpening, pattern identification, restoration, and retrieval. Mathematically future research can be carried out with the results of Fekete inequality obtained for inverse functions and can be applied in image processing.

## Data Availability

Image source file for Canny image is given below: Closed PalmThe MATLAB code used in this investigation are accessible through the following link MATLAB CODE-Mendeley Data BHASKARA, SRUTHAKEERTHI; H, Priya, “Texture analysis using Horadam Polynomial coefficient estimate for the class of Sakaguchi kind function”, Mendeley Data, V1, doi: 10.17632/pw779rf4jn.1.
